# Continuum of spin excitations in an ordered magnet

**DOI:** 10.1016/j.xinn.2024.100769

**Published:** 2025-01-17

**Authors:** Jieming Sheng, Le Wang, Wenrui Jiang, Han Ge, Nan Zhao, Tiantian Li, Maiko Kofu, Dehong Yu, Wei Zhu, Jia-Wei Mei, Zhentao Wang, Liusuo Wu

**Affiliations:** 1 Department of Physics, Southern University of Science and Technology, Shenzhen 518055, China; 2 School of Physical Sciences, Great Bay University and Great Bay Institute for Advanced Study, Dongguan 523000, China; 3 Shenzhen Institute for Quantum Science and Engineering, Southern University of Science and Technology, Shenzhen 518055, China; 4 International Quantum Academy, Shenzhen 518048, China; 5 J-PARC Center, Japan Atomic Energy Agency, Tokai, Ibaraki 319-1195, Japan; 6 Australian Nuclear Science and Technology Organisation, Lucas Heights, NSW 2234, Australia; 7 School of Science, Westlake University, Hangzhou 310030, China; 8 Institute of Natural Sciences, Westlake Institute of Advanced Study, Hangzhou 310024, China; 9 Shenzhen Key Laboratory of Advanced Quantum Functional Materials and Devices, Southern University of Science and Technology, Shenzhen 518055, China; 10 Center for Correlated Matter and School of Physics, Zhejiang University, Hangzhou 310058, China

**Keywords:** frustrated magnets, spin excitation continuum, quantum spin liquid, neutron scattering

## Abstract

Spin excitation continua observed in neutron scattering studies are often considered to be strong evidence of quantum spin liquid formation. In a disorder-free magnetic compound with a quantum spin liquid ground state, the elementary excitation is no longer the conventional spin waves (magnons). Instead, the magnons fractionalize into spinons, producing a characteristic two-spinon continuum. However, it remained uncertain whether a clean, ordered antiferromagnet could exhibit a continuous spectrum similar to that of a quantum spin liquid. This paper presents evidence of a spin excitation continuum in the magnetically ordered state of Na_2_BaCo(PO_4_)_2_, where free spinons are absent. This challenges the interpretation of such a continuum as a definitive signature of a quantum spin liquid in new material studies.

## Public summary

• Inelastic neutron scattering experiments performed across all low-temperature phases in Na_2_BaCo(PO_4_)_2_.
• Density matrix renormalization group calculations agree quantitatively with neutron scattering results.
• Spin excitation continua induced by quantum fluctuations, even in the presence of magnetic ordered states.
• Spin excitation continuum alone should not be used as “smoking gun evidence” of quantum spin liquids.

## Graphical abstract

**Figure:**
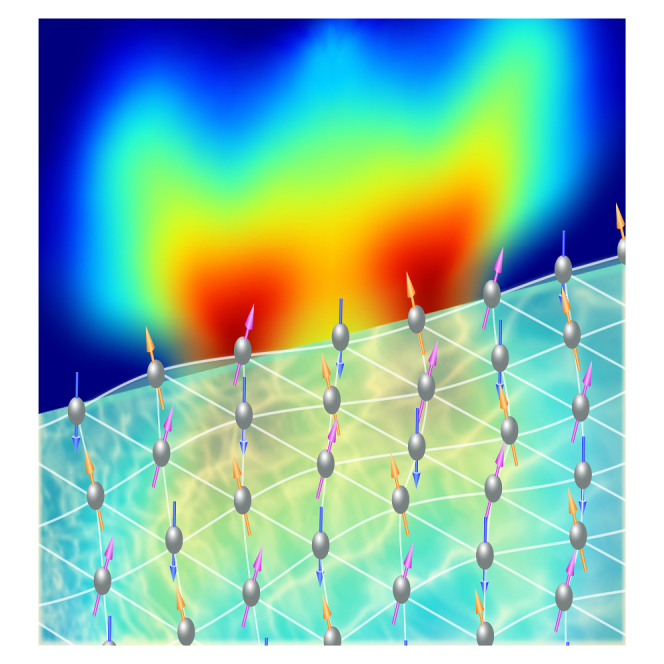

## Introduction

The quantum spin liquid (QSL) state was originally proposed by Philip W. Anderson to describe the ground state of the spin-1/2 triangular lattice (TL) Heisenberg model, where geometric frustration and quantum fluctuation prevent long-range magnetic ordering at zero temperature.^1^ The QSL, if realized, could serve as a strong foundation for fault-tolerant quantum computation^2^^,^^3^ and significantly enhance our understanding of unconventional superconductivity mechanisms.^4^^,^^5^ Tremendous efforts have been devoted over the years to realizing QSLs in real materials.^6^^,^^7^^,^^8^^,^^9^

While “there is no single experimental feature that identifies a spin-liquid state,”^6^ several classes of materials have emerged as QSL candidates, including the organic compounds (*κ*-(BEDT-TTF)_2_Cu_2_(CN)_3_,^10^^,^^11^ EtMe_3_Sb[Pd(dmit)_2_]_2_,^12^^,^^13^^,^^14^^,^^15^ and *κ*-H_3_(Cat-EDT-TTF)_2_^16^) and inorganic compounds on Kagome (ZnCu_3_(OH)_6_Cl_2_^17^), triangular (YbZn_2_GaO_5_,^18^ NdTa_7_O_19_,^19^ NaRuO_2_,^20^^,^^21^ rare-earth chalcogenides NaYb*X*_2_, where *X* = {O, S, Se},^22^^,^^23^^,^^24^^,^^25^^,^^26^^,^^27^^,^^28^^,^^29^ and 1T-TaS_2_^30^), honeycomb (*α*-RuCl_3_^31^^,^^32^^,^^33^^,^^34^ and BaCo_2_(AsO_4_)_2_^35^^,^^36^^,^^37^^,^^38^), distorted bilayer Kagome (Ca_10_Cr_7_O_28_^39^^,^^40^), hyper-Kagome (Na_4_Ir_3_O_8_^41^), and trillium (K_2_Ni_2_(SO_4_)_3_^42^^,^^43^^,^^44^) lattices.

In QSLs, conventional spin wave excitations fractionalize into the spinon continuum,^5^^,^^7^^,^^45^^,^^46^ observable through inelastic neutron scattering (INS) experiments. Indeed, several QSL candidates exhibit continuous excitation spectra,^17^^,^^24^^,^^27^^,^^28^^,^^29^^,^^33^^,^^37^^,^^39^^,^^40^^,^^43^^,^^44^ though the precise origins of these continua remain debated. For example, the disorder may influence continuum formation in ZnCu_3_(OH)_6_Cl_2_ and NaYb*X*_2_ (*X* = O, S, Se).

Thus, it is worth considering whether the observed INS continuum can be directly linked to QSL behavior for materials that remain disordered at the lowest available temperatures. For compounds with additional active degrees of freedom (e.g., charge) or disorder, drawing definite conclusions is difficult, as magnon lifetimes can be significantly reduced by scattering with electrons or disorder. Nevertheless, we can still ask, for good insulators without intrinsic disorder, do fully continuous excitations always signify the QSL state?

This report highlights the spin-1/2 TL antiferromagnet Na_2_BaCo(PO_4_)_2_^47^ as an excellent material for addressing this long-standing question. Na_2_BaCo(PO_4_)_2_ was initially investigated as a QSL candidate based on thermodynamic measurements down to 50 mK at zero field^47^^,^^48^ until a transition temperature *T*_N_ of ∼150 mK was identified, marking the onset of an antiferromagnetic (AFM) state.^49^^,^^50^ As we shall demonstrate later, while Na_2_BaCo(PO_4_)_2_ displays a distinct long-range magnetic order at low temperatures, it also exhibits a spin excitation continuum. Our experimental and theoretical analyses reveal that the continuum arises intrinsically from the spin-1/2 XXZ model on the TL rather than from disorder effects. These findings reveal that a spin excitation continuum can coexist with long-range magnetic order due to the interplay of geometric frustration and quantum fluctuations.

## Results and discussion

The Hamiltonian of Na_2_BaCo(PO_4_)_2_ was determined through extensive thermodynamic and spectroscopic measurements,^50^ leading to a spin-1/2 XXZ model on the TL:

(Equation 1) $$H=J\sum_{\left\langle ij\right\rangle }\left(S_{i}^{x}S_{j}^{x}+S_{i}^{y}S_{j}^{y}+\Delta S_{i}^{z}S_{j}^{z}\right)-g_{c}\mu_{B}B\sum_{i}S_{i}^{z}\text{,}$$

where *J* = 0.076(1) meV is the nearest-neighbor interaction of the TL, Δ = 1.645(1) is the exchange anisotropy, *g*_c_ is the *g* factor along the c axis, and $\mu_{B}$ is the Bohr magneton. A magnetic field *B* applied along the c axis couples to the $S_{i}^{z}$ component in Equation 1 via the Zeeman effect. Interlayer interactions are at least ten times weaker than *J*, validating the two-dimensional TL as the primary model for most analyses in this report (see Figure S2). Other symmetry-allowed exchange anisotropies were found to be negligible compared to *J*.^50^ Independent model extraction via the tensor network^51^^,^^52^ has yielded very similar parameters.

**Figure 1:**
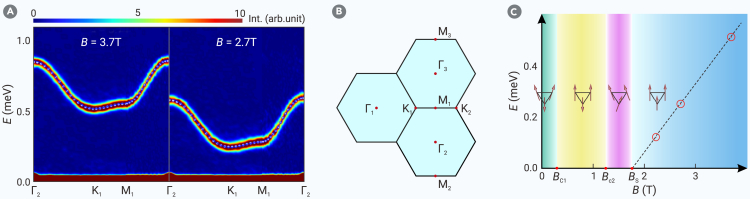
Single-magnon BEC in the fully polarized phase (A) Inelastic neutron scattering results of Na_2_BaCo(PO_4_)_2_ for *B* = 3.7 T and *B* = 2.7 T at 60 mK. The empty black circles are centers of the spin wave fitted by a Gaussian function. The solid black line is the theoretical 1-magnon dispersion (Equation 2) with *J* = 0.076 meV, Δ = 1.645, and *g*_c_ = 4.645. (B) High-symmetry momentum points used in the trajectory of (A), where Γ_1_ = (0*,*0*,*0) is the center of the first Brillouin zone. (C) The red empty circles are the measured spin wave gap at K point. The dashed black line is the theoretical value given by Equation 2. The *T* = 0 phases are schematically indicated along with the critical magnetic fields {*B*_c1_, *B*_c2_, *B*_s_}. The softening of the spin wave gap at *B*_s_ is a signature of magnon Bose–Einstein condensation (BEC). Error bars denote standard deviations in (A).

We start by demonstrating that the magnetic exchanges in Na_2_BaCo(PO_4_)_2_ are pure nearest-neighbor XXZ type. By fully polarizing the magnetic moments along the c axis with a strong magnetic field *B* > *B*_s_, the linear spin wave (LSW) theory becomes an exact solution, enabling precise model extraction. As shown in Figures 1A and 1B, the sharp spin waves agree perfectly with the theoretical dispersion

(Equation 2) $$\omega_{k}^{\left(\text{FP}\right)}=2JS\left(\cos\;k_{x}+2\;\cos\frac{k_{x}}{2}\cos\frac{\sqrt{3}k_{y}}{2}\right)-6S\Delta J+g_{c}\mu_{B}B\text{,}$$

where the magnetic moment is *S* = 1/2. Clearly, structural and magnetic disorders are negligible in this compound; otherwise, the spin waves above *B*_s_ would broaden due to magnon-disorder scattering.

Using multiple magnetic fields above *B*_s_ (Figure 1), we determined the *g* factor to be *g*_c_ = 4.645(22). Notably, the 1-magnon gap at the K point decreases linearly to zero and condenses precisely at the quantum critical point (QCP) *B* = *B*_s_ (Figure 1C), indicating that the QCP corresponds to a 1-magnon Bose–Einstein condensation (BEC).^53^ The ground state just below *B*_s_ can be readily inferred from the 1-magnon state being condensed: it is both a superfluid due to magnon condensation and a solid due to translational symmetry breaking (softened at the K point). In other words, this phase is a supersolid in the bosonic representation.^54^^,^^55^^,^^56^ In the spin representation, cluster mean field and density matrix renormalization group (DMRG) calculations identified this supersolid phase as having a “V” shape.^57^^,^^58^ We note that this is drastically different from the spin-1 compound Na_2_BaNi(PO_4_)_2_, where the 2-magnon bound state condenses at *B*_s_, while the 1-magnon remains gapped at the QCP.^59^

After confirming that the TL XXZ model (Equation 1) accurately describes Na_2_BaCo(PO_4_)_2_ and ruling out disorder effects, we now investigate the impact of quantum fluctuations, which become significant at lower fields. Due to the small saturation field of Na_2_BaCo(PO_4_)_2_ (*B*_s_ = 1.8 T with B∥c), our comprehensive INS measurements fully mapped the magnetic ground states of this easy-axis TL XXZ model under a field along the c axis, including the Y, up-up-down (UUD), V, and fully polarized (FP) phases (see Figure 1C). The calculated static spin structure factors for these phases are presented in Figure S7.

**Figure 2:**
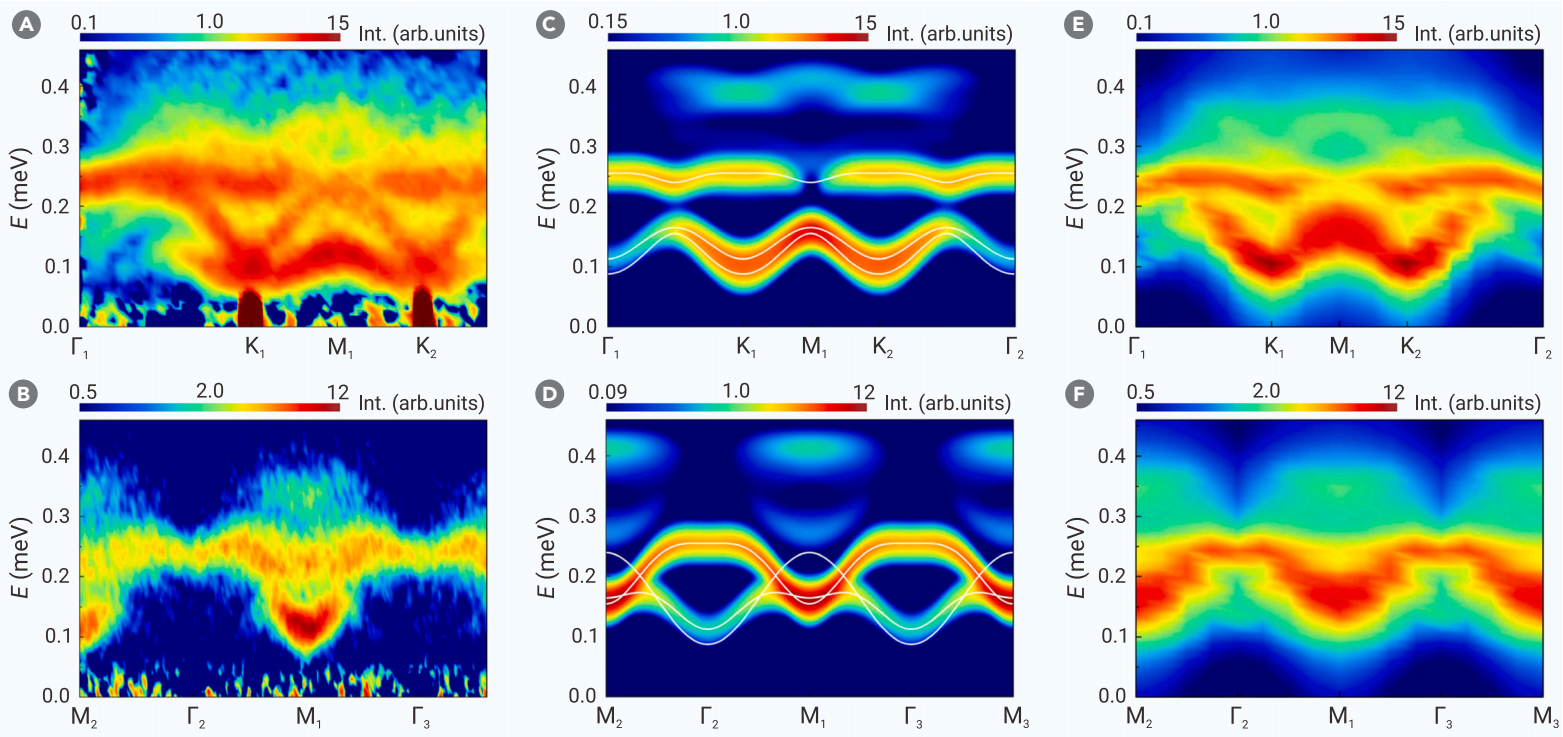
Spin excitation spectra along the high-symmetry momentum directions at *B* = 0.75 T (UUD phase) (A and B) Inelastic neutron scattering results at *T* = 60 mK with a 60 mK-3.7 T dataset subtracted as background. The incident neutron energy is *E*_*i*_ = 2.63 meV. (C and D) *T* = 0 dynamic spin structure factor S(k,ω) from linear spin wave, including both the 1-magnon and 2-magnon contributions. The solid white lines are the 1-magnon dispersions. (E and F) *T* = 0 dynamic spin structure factor S(k,ω) from density matrix renormalization group on 6-leg cylinder.

The UUD phase corresponds to the 1/3-magnetization plateau within 0.3 T < *B* < 1.2 T. To investigate its magnetic excitation, we conducted INS measurements at *B* = 0.75 T and *T* = 60 mK (see Figures 2A and 2B). LSW calculations (see supplemental information) are shown in Figures 2C and 2D for comparison. The key features, including three gapped magnon branches with a global minimum at K points, are qualitatively captured by LSW. However, LSW fails to accurately reproduce certain features. For example, the highest spin wave branch from measurements rises when moving from the Γ_1_ point toward the K_1_ direction (Figure 2A), contrary to the LSW prediction (Figure 2C). Additionally, the 2-magnon continuum from INS measurements, primarily between 0.3 and 0.4 meV, shows slight discrepancies compared to LSW calculations.

To assess whether such discrepancies arise from model inaccuracies or quantum fluctuation effects, an unbiased approach is needed to evaluate how well the model (Equation 1) describes the spin excitation spectra of Na_2_BaCo(PO_4_)_2_. This is particularly important when magnon interactions are strong or when magnons inadequately represent the system’s elementary quasiparticles. We employed the DMRG to compute the dynamic spin structure factor.^60^^,^^61^ As shown in Figures 2E and 2F, DMRG indeed refines the LSW spectrum to align more closely with experimental results. The initial slope of the highest magnon branch near the Γ point is corrected. The 2-magnon continuum in the LSW results shifts downward, aligning closely with the INS results. The middle magnon branch near the M point shifts upward, becoming consistent with the INS results, though it remains unresolved from the lowest branch within our resolution (Figure 2E).

The comparison between the experimental and theoretical results of the UUD phase reveals significant insights. Firstly, the nearest-neighbor XXZ model (Equation 1) is confirmed as an accurate microscopic model for Na_2_BaCo(PO_4_)_2_. Consistent with the INS results in the FP phase, the absence of disorder in our compound produces sharp 1-magnon excitations in the UUD phase. Secondly, quantum fluctuations significantly influence the spin excitation spectrum, not only renormalizing the 1-magnon dispersion but also transferring substantial spectral weight to the 2-magnon continuum. Although the 2-magnon continuum is present, it does not cause any noticeable decay of the 1-magnon branches. This is expected, as the collinear UUD state lacks the 3-magnon interaction necessary for 1- to 2-magnon decays.^62^^,^^63^ Lastly, we note that a standard LSW can cause systematic error in fitting the 1-magnon bands in the UUD phase due to finite higher-order terms. For more accurate results, one should either fit the bands in the FP phase or employ more accurate methods, such as DMRG, when *B*_s_ is large.

**Figure 3:**
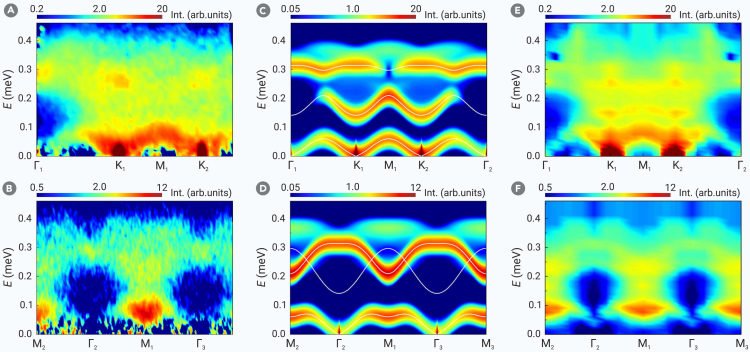
Spin excitation spectra along the high-symmetry momentum directions at *B* = 1.2 T (V phase) (A and B) Inelastic neutron scattering results at *T* = 60 mK with a 60 mK-3.7 T dataset subtracted as background. The incident neutron energy is *E*_*i*_ = 2.63 meV. (C and D) *T* = 0 dynamic spin structure factor S(k,ω) from linear spin wave, including both the 1-magnon and 2-magnon contributions. The solid white lines are the 1-magnon dispersions. (E and F) *T* = 0 dynamic spin structure factor S(k,ω) from density matrix renormalization group on 6-leg cylinder.

Unlike the UUD phase with its gapped spin excitations, the high-field V and low-field Y phases exhibit Goldstone modes from spontaneous *U*(1) symmetry breaking. More importantly, the condition of zero 3-magnon interactions in the UUD state no longer applies in these noncollinear states, allowing the single-to 2-magnon decay channel. Figure 3 shows both the experimental and theoretical spin excitation spectra in the V phase at 1.2 T and 60 mK. The Goldstone mode at K points remains sharply defined, while higher-energy modes from INS (Figures 3A and 3B) are notably broadened, possibly due to decay into the 2-magnon continuum. Indeed, the LSW results reveal substantial overlap between the higher-energy modes and the 2-magnon continuum (Figure 3), enabling this decay channel. Again, the DMRG results quantitatively agree with the INS findings, confirming that the broadening is an intrinsic feature of the TL XXZ model and not due to disorder.

**Figure 4:**
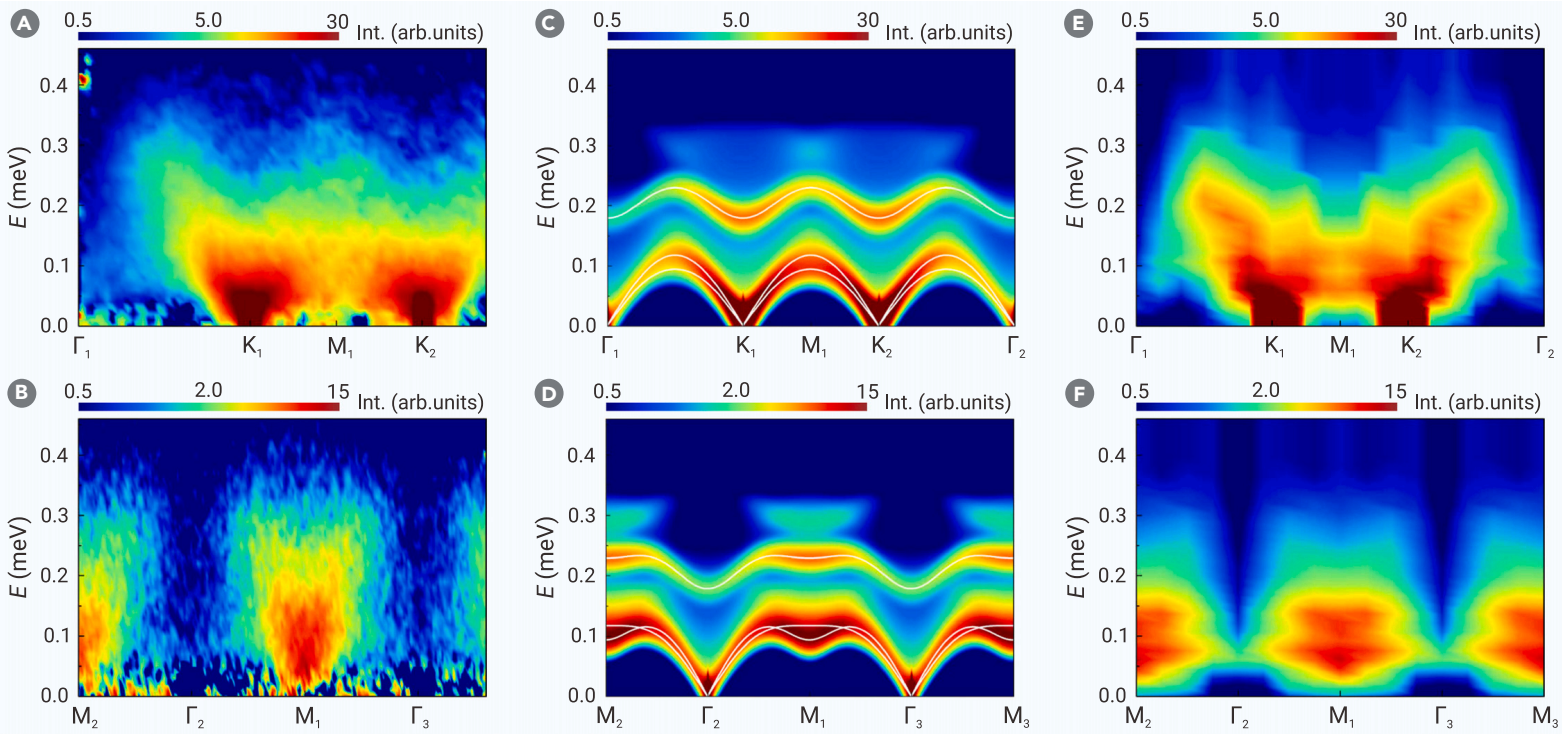
Spin excitation spectra along the high-symmetry momentum directions at *B* = 0 T (Y phase) (A and B) Inelastic neutron scattering results at *T* = 60 mK with a 60 mK-3.7 T dataset subtracted as background. The incident neutron energy is *E*_*i*_ = 2.63 meV. (C and D) *T* = 0 dynamic spin structure factor S(k,ω) from linear spin wave, including both the 1-magnon and 2-magnon contributions. The solid white lines are the 1-magnon dispersions. (E and F) *T* = 0 dynamic spin structure factor S(k,ω) from density matrix renormalization group on 6-leg cylinder.

Finally, the zero-field INS results are shown in Figures 4A and 4B, measured at *T* = 60 mK, well below the Néel temperature *T*_N_ = 150 mK, where the Y phase develops long-range order. Typically, a magnetically ordered state would exhibit some distinct spin waves. Surprisingly, Figures 4A and 4B show practically no sharply defined spin waves, with only a continuum of spin excitations visible. This continuum was initially observed on PELICAN at ANSTO with an incident energy *E*_*i*_ = 3.7 meV and an energy resolution of 0.13 meV (see Figure S1). We subsequently verified this continuum on AMATERAS at J-PARC with improved energy resolution of 0.047 meV and *E*_*i*_ = 2.63 meV. Additional spin excitation spectra are presented in Figures S3–S6.

Without the prior comprehensive analysis confirming long-range magnetic order and ruling out disorder effects, the observed continuum might have been misinterpreted as “smoking-gun evidence” for a QSL. However, the QSL proposal is clearly invalid for Na_2_BaCo(PO_4_)_2_, and the spin wave broadening appears to be inherent to the TL XXZ model in the ordered Y phase.

In fact, the LSW reveals that all the 1-magnon branches strongly overlap with the 2-magnon continuum (Figures 4C and 4D), indicating magnon instability throughout the decay process. This is confirmed by the DMRG results (Figures 4E and 4F), where sharp spin waves decay into a continuum of spin excitations, consistent with the INS measurements.

Strong quantum fluctuations, evidenced by the agreement between the INS and DMRG results, suggest that the semiclassical magnon-based framework may no longer be adequate. While *free* spinons are not permitted in the long-range-ordered state, spinon pairs can, in principle, bind to form magnons while retaining their internal two-spinon structure.^64^^,^^65^^,^^66^ This framework is likely relevant for the TL spin-1/2 models, where a tiny next-nearest-neighbor interaction (approximately 6% of the nearest-neighbor coupling) can induce a QSL phase in the Heisenberg limit (Δ → 1),^67^^,^^68^ characterized by free spinons on the QSL side of the QCP. Notably, the Schwinger boson formalism incorporating this bound-spinon perspective effectively captures both sharp and broad spin excitations in Ba_3_CoSb_2_O_9_.^64^^,^^65^^,^^66^^,^^69^ For Na_2_BaCo(PO_4_)_2_, the QSL on the other side of the putative QCP is argued to be of the Dirac type.^70^

Recent studies have shown that a few other TL compounds, such as AYb*X*_2_ series, also exhibit similar continuous spin excitation spectra.^27^^,^^29^^,^^71^^,^^72^ While similar physics may explain these continua, we contend that Na_2_BaCo(PO_4_)_2_ is better suited for exploring the intrinsic quantum fluctuations responsible for continuum formation. Notably, neither NaYbSe_2_ nor CsYbSe_2_ exhibits true long-range order down to the lowest available temperature; while KYbSe_2_ shows a specific heat anomaly near 290 mK, neutron diffraction does not detect magnetic Bragg peaks along the *L* direction below this temperature.^29^ Similarly, in the spin-5/2 compound Ba_3_MnSb_2_O_9_, a broad spectrum appears in the long-range-ordered state, with potential causes such as thermal fluctuations, domain effect, and 4% Sb deficiency.^73^ Thus, these systems are not ideal for isolating quantum fluctuations as the sole origin of the observed continua in a long-range-ordered state. In future studies on Na_2_BaCo(PO_4_)_2_, it would be interesting to see if a controlled disorder can be introduced and how it affects both the magnetic ground state and the excitation spectra.

We note that the spin excitation spectra obtained through INS and similar techniques are inherently limited by instrumental resolution. Therefore, any claim on observing a spin excitation continuum must clearly specify the resolution used, regardless of the continuum’s origin (e.g., free spinon, magnon decay). In this work, the highest INS resolution achieved is 0.047 meV, about an order of magnitude smaller than the spin excitation spectrum bandwidth of Na_2_BaCo(PO_4_)_2_ at *B* = 0 T. While this resolution cannot entirely rule out the presence of remaining sharp magnon modes near the continuum edge, it allows us to conclude that the zero-field spectrum is predominantly continuum dominated. Additionally, small terms such as interlayer interactions, off-diagonal exchange anisotropies, and further-neighbor interactions are negligible at the current resolution but may become significant with higher-resolution INS measurements. In other words, Na_2_BaCo(PO_4_)_2_ could exhibit deviation from a perfect nearest-neighbor XXZ model under higher-resolution scrutiny, which would be an intriguing avenue for future research.

## Conclusion

To summarize, we have performed INS and theoretical analysis for Na_2_BaCo(PO_4_)_2_, uncovering the role of quantum fluctuations in the spin excitation spectra of long-range magnetically ordered phases. Notably, significant spectral weight shifts from the 1-magnon excitation to the 2-magnon continuum, with strong magnon decay under kinematic condition. The spin wave broadening is particularly pronounced in the long-range-ordered Y phase, where only a continuum of excitation is observed with an INS resolution of 0.047 meV. This finding challenges conventional practice in the field that often perceives such a continuum as a signature of the QSL—as we have shown, such features can also arise in systems with long-range magnetic order exhibiting strong quantum fluctuations.

## Materials and methods

### Materials synthesis

Single crystals of Na_2_BaCo(PO_4_)_2_ used in this study were grown using the NaCl-flux method. The starting materials, Na_2_CO_3_ (Alfa, 99.5%), BaCO_3_ (Alfa, 99.95%), CoO (Aladdin, 99%), (NH_4_)_2_HPO_4_ (Aladdin, 99.99%), and NaCl (Alfa, 99.99%), were thoroughly ground together in a molar ratio of 1:1:1:2:5. The mixture was then loaded into an alumina crucible with a lid and heated to 850°C for 20 h, followed by a slow cooling process to 750°C over 100 h. After soaking the product in water to remove residual NaCl, numerous hexagonal pink single crystals were mechanically separated from the walls of the crucible.

### Neutron scattering

Neutron scattering experiments were conducted with the cold-neutron disk chopper spectrometer AMATERAS (BL14 beamline) with fixed incident energy *E*_*i*_ = 2.63 meV (energy resolution is about 0.047 meV) at the Materials and Life Science Experimental Facility (MLF), J-PARC,^74^ and the time-of-flight cold neutron spectrometer PELICAN at the OPAL reactor ANSTO with a fixed incident energy *E*_*i*_ = 3.71 meV (energy resolution is about 0.13 meV). The INS results presented in the main text are from AMATERAS, and the ones from PELICAN are shown in the supplemental information for comparison. Hundreds of Na_2_BaCo(PO_4_)_2_ single crystals were co-aligned on the oxygen-free copper sheets for the INS experiments, with a total mass of approximately 3 g. The samples were cooled using a dilution refrigerator insert in a 7 T magnet on both spectrometers, with the magnetic field applied along the c axis. The INS data were collected at temperatures of 60 and 450 mK with different magnetic fields and processed using the freely available Utsusemi^75^ and Dave software tool.^76^

## Data and code availability

All data necessary to evaluate the conclusions of the paper are included in this report and its supplemental information. Additional data and computer codes can be made available by the corresponding authors upon reasonable request.

## Acknowledgments

We thank Gang Chen for helpful discussions and G. Davidson for the great support in setting up and operating the superconducting magnet and the dilution insert throughout the experiment on Pelican. The research was supported by the 10.13039/501100012166National Key Research and Development Program of China (grant nos. 2021YFA1400400, 2022YFA1402204, and 2024YFA1408303), the 10.13039/501100001809National Natural Science Foundation of China (grant nos. 12134020, 12104255, 12204223, 12374124, and 12374146), the Open Fund of the China Spallation Neutron Source Songshan Lake Science City (grant no. KFKT2023A06), the 10.13039/501100012226Fundamental Research Funds for the Central Universities (grant no. 226-2024-00068), and the Guangdong Provincial Quantum Science Strategic Initiative (grant nos. GDZX2401006 and GDZX2401007). The authors also acknowledge the neutron beam time awarded by the Materials and Life Science Experimental Facility of the Japan Proton Accelerator Research Complex (J-PARC) through proposal no. 2021B0185 and Australia’s Nuclear Science and Technology Organisation (ANSTO) through proposal no. P9457. The funders had no role in the study design, data collection and analysis, decision to publish, or preparation of the manuscript.

## Author contributions

J.S., L. Wu, Z.W., and J.-W.M. designed the experiments. W.J., L. Wang, and J.-W.M. provided the single crystals used in this study. J.S., M.K., L. Wu, and D.Y. carried out the neutron scattering experiments. Z.W. carried out the LSW calculations and developed the theoretical explanations. W.Z. performed the DMRG calculations. H.G., N.Z., and T.L. carried out the low-temperature measurements. All authors contributed to and approved the manuscript.

## Declaration of interests

The authors declare no competing interests.

## Lead contact website

https://www.sustech.edu.cn/en/faculties/wuliusuo.html

## Supplemental information

Document S1. Figures S1–S7 and supplemental materials and methods

Document S2. Article plus supplemental information

## References

[1] Anderson P.W. (1973). Resonating valence bonds: A new kind of insulator?. Mater. Res. Bull..

[2] Kitaev A. (2003). Fault-tolerant quantum computation by anyons. Ann. Phys..

[3] Kitaev A. (2006). Anyons in an exactly solved model and beyond. Ann. Phys..

[4] Anderson P.W. (1987). The Resonating Valence Bond State in La₂CuO₄ and Superconductivity. Science.

[5] Lee P.A., Nagaosa N., Wen X.G. (2006). Doping a Mott insulator: Physics of high-temperature superconductivity. Rev. Mod. Phys..

[6] Balents L. (2010). Spin liquids in frustrated magnets. Nature.

[7] Zhou Y., Kanoda K., Ng T.K. (2017). Quantum spin liquid states. Rev. Mod. Phys..

[8] Wen J., Yu S.L., Li S. (2019). Experimental identification of quantum spin liquids. npj Quantum Mater..

[9] Broholm C., Cava R.J., Kivelson S.A. (2020). Quantum spin liquids. Science.

[10] Shimizu Y., Miyagawa K., Kanoda K. (2003). Spin Liquid State in an Organic Mott Insulator with a Triangular Lattice. Phys. Rev. Lett..

[11] Yamashita S., Nakazawa Y., Oguni M. (2008). Thermodynamic properties of a spin-1/2 spin-liquid state in a κ-type organic salt. Nat. Phys..

[12] Itou T., Oyamada A., Maegawa S. (2008). Quantum spin liquid in the spin-1∕2 triangular antiferromagnet EtMe₃Sb[Pd(dmit)₂]₂. Phys. Rev. B.

[13] Yamashita M., Nakata N., Senshu Y. (2010). Highly Mobile Gapless Excitations in a Two-Dimensional Candidate Quantum Spin Liquid. Science.

[14] Bourgeois-Hope P., Laliberté F., Lefrançois E. (2019). Thermal Conductivity of the Quantum Spin Liquid Candidate EtMe₃Sb[Pd(dmit)₂]₂: No Evidence of Mobile Gapless Excitations. Phys. Rev. X.

[15] Ni J.M., Pan B.L., Song B.Q. (2019). Absence of Magnetic Thermal Conductivity in the Quantum Spin Liquid Candidate EtMe₃Sb[Pd(dmit)₂]₂. Phys. Rev. Lett..

[16] Isono T., Kamo H., Ueda A. (2014). Gapless Quantum Spin Liquid in an Organic Spin-1/2 Triangular-Lattice κ-H₃(Cat-EDT-TTF)₂. Phys. Rev. Lett..

[17] Han T.H., Helton J.S., Chu S. (2012). Fractionalized excitations in the spin-liquid state of a kagome-lattice antiferromagnet. Nature.

[18] Bag R., Xu S., Sherman N.E. (2024). Evidence of Dirac Quantum Spin Liquid in YbZn₂GaO₅.. Phys. Rev. Lett..

[19] Arh T., Sana B., Pregelj M. (2022). The Ising triangular-lattice antiferromagnet neodymium heptatantalate as a quantum spin liquid candidate. Nat. Mater..

[20] Ortiz B.R., Sarte P.M., Avidor A.H. (2023). Quantum disordered ground state in the triangular-lattice magnet NaRuO₂. Nat. Phys..

[21] Ma J. (2023). Spins don’t align here. Nat. Phys..

[22] Liu W., Zhang Z., Ji J. (2018). Rare-Earth Chalcogenides: A Large Family of Triangular Lattice Spin Liquid Candidates. Chin. Phys. Lett..

[23] Baenitz M., Schlender P., Sichelschmidt J. (2018). NaYbS₂: A planar spin-1/2 triangular-lattice magnet and putative spin liquid. Phys. Rev. B.

[24] Bordelon M.M., Kenney E., Liu C. (2019). Field-tunable quantum disordered ground state in the triangular-lattice antiferromagnet NaYbO₂. Nat. Phys..

[25] Ding L., Manuel P., Bachus S. (2019). Gapless spin-liquid state in the structurally disorder-free triangular antiferromagnet NaYbO₂. Phys. Rev. B.

[26] Sarkar R., Schlender P., Grinenko V. (2019). Quantum spin liquid ground state in the disorder free triangular lattice NaYbS₂. Phys. Rev. B.

[27] Dai P.L., Zhang G., Xie Y. (2021). Spinon Fermi Surface Spin Liquid in a Triangular Lattice Antiferromagnet NaYbSe₂. Phys. Rev. X.

[28] Wu J., Li J., Zhang Z. (2022). Magnetic field effects on the quantum spin liquid behaviors of NaYbS₂. Quantum Front..

[29] Scheie A.O., Kamiya Y., Zhang H. (2024). Nonlinear magnons and exchange Hamiltonians of the delafossite proximate quantum spin liquid candidates KYbSe₂ and NaYbSe₂. Phys. Rev. B.

[30] Law K.T., Lee P.A. (2017). 1T-TaS₂ as a quantum spin liquid. Proc. Natl. Acad. Sci. USA.

[31] Wang Z., Reschke S., Hüvonen D. (2017). Magnetic Excitations and Continuum of a Possibly Field-Induced Quantum Spin Liquid in α-RuCl₃. Phys. Rev. Lett..

[32] Zheng J., Ran K., Li T. (2017). Gapless Spin Excitations in the Field-Induced Quantum Spin Liquid Phase of α-RuCl₃. Phys. Rev. Lett..

[33] Banerjee A., Lampen-Kelley P., Knolle J. (2018). Excitations in the field-induced quantum spin liquid state of α-RuCl₃. npj Quantum Mater..

[34] Kasahara Y., Ohnishi T., Mizukami Y. (2018). Majorana quantization and half-integer thermal quantum Hall effect in a Kitaev spin liquid. Nature.

[35] Ferrenti A.M., Siegler M.A., Ghosh S. (2022). Chemical tuning of a honeycomb magnet through a critical point. arXiv.

[36] Tu C., Dai D., Zhang X. (2022). Evidence for gapless quantum spin liquid in a honeycomb lattice. arXiv.

[37] Zhang X., Xu Y., Halloran T. (2023). A magnetic continuum in the cobalt-based honeycomb magnet BaCo₂(AsO₄)₂. Nat. Mater..

[38] Halloran T., Desrochers F., Zhang E.Z. (2023). Geometrical frustration versus Kitaev interactions in BaCo₂(AsO₄)₂. Proc. Natl. Acad. Sci. USA.

[39] Balz C., Lake B., Reuther J. (2016). Physical realization of a quantum spin liquid based on a complex frustration mechanism. Nat. Phys..

[40] Balz C., Lake B., Nazmul Islam A.T.M. (2017). Magnetic Hamiltonian and phase diagram of the quantum spin liquid Ca₁₀Cr₇O₂₈. Phys. Rev. B.

[41] Okamoto Y., Nohara M., Aruga-Katori H. (2007). Spin-Liquid State in the *S*=1/2 Hyperkagome Antiferromagnet Na₄Ir₃O₈. Phys. Rev. Lett..

[42] Živković I., Favre V., Salazar M.C. (2021). Magnetic Field Induced Quantum Spin Liquid in the Two Coupled Trillium Lattices of K₂Ni₂(SO₄)₃. Phys. Rev. Lett..

[43] Gonzalez M.G., Noculak V., Sharma A. (2024). Dynamics of K₂2Ni₂(SO₄)₃ governed by proximity to a 3D spin liquid model. Nat. Commun..

[44] Yao W., Huang Q., Xie T. (2023). Continuous Spin Excitations in the Three-Dimensional Frustrated Magnet K₂Ni₂(SO₄)₃. Phys. Rev. Lett..

[45] Baskaran G., Zou Z., Anderson P.W. (1987). The resonating valence bond state and high-Tc superconductivity - A mean field theory. Solid State Commun..

[46] Zhu W., Gong S.S., Sheng D.N. (2019). Identifying spinon excitations from dynamic structure factor of spin-1/2 Heisenberg antiferromagnet on the Kagome lattice. Proc. Natl. Acad. Sci. USA.

[47] Zhong R., Guo S., Xu G. (2019). Strong quantum fluctuations in a quantum spin liquid candidate with a Co-based triangular lattice. Proc. Natl. Acad. Sci. USA.

[48] Lee S., Lee C.H., Berlie A. (2021). Temporal and field evolution of spin excitations in the disorder-free triangular antiferromagnet Na_2_BaCo(PO_4_)_2_. Phys. Rev. B.

[49] Li N., Huang Q., Yue X.Y. (2020). Possible itinerant excitations and quantum spin state transitions in the effective spin-1/2 triangular-lattice antiferromagnet Na₂BaCo(PO₄)₂. Nat. Commun..

[50] Sheng J., Wang L., Candini A. (2022). Two-dimensional quantum universality in the spin-1/2 triangular-lattice quantum antiferromagnet Na₂BaCo(PO₄)₂. Proc. Natl. Acad. Sci. USA.

[51] Gao Y., Fan Y.C., Li H. (2022). Spin supersolidity in nearly ideal easy-axis triangular quantum antiferromagnet Na₂BaCo(PO₄)₂. npj Quantum Mater..

[52] Xiang J., Zhang C., Gao Y. (2024). Giant magnetocaloric effect in spin supersolid candidate Na₂BaCo(PO₄)₂. Nature.

[53] Zapf V., Jaime M., Batista C.D. (2014). Bose-Einstein condensation in quantum magnets. Rev. Mod. Phys..

[54] Andreev A.F., Lifshitz I.M. (1969). Quantum Theory of Defects in Crystals. Zh. Eksp. Teor. Fiz..

[55] Chester G.V. (1970). Speculations on Bose-Einstein Condensation and Quantum Crystals. Phys. Rev. A.

[56] Leggett A.J. (1970). Can a Solid Be “Superfluid”?. Phys. Rev. Lett..

[57] Yamamoto D., Marmorini G., Danshita I. (2014). Quantum Phase Diagram of the Triangular-Lattice *XXZ* Model in a Magnetic Field. Phys. Rev. Lett..

[58] Sellmann D., Zhang X.F., Eggert S. (2015). Phase diagram of the antiferromagnetic XXZ model on the triangular lattice. Phys. Rev. B.

[59] Sheng J., Mei J.W., Wang L. (2025). Bose-Einstein condensation of a two-magnon bound state in a spin-1 triangular lattice.. Nat. Mater..

[60] Kühner T.D., White S.R. (1999). Dynamical correlation functions using the density matrix renormalization group. Phys. Rev. B.

[61] Jeckelmann E. (2002). Dynamical density-matrix renormalization-group method. Phys. Rev. B.

[62] Chernyshev A.L., Zhitomirsky M.E. (2006). Magnon Decay in Noncollinear Quantum Antiferromagnets. Phys. Rev. Lett..

[63] Zhitomirsky M.E., Chernyshev A.L. (2013). Colloquium: Spontaneous magnon decays. Rev. Mod. Phys..

[64] Ghioldi E.A., Gonzalez M.G., Zhang S.S. (2018). Dynamical structure factor of the triangular antiferromagnet: Schwinger boson theory beyond mean field. Phys. Rev. B.

[65] Zhang S.S., Ghioldi E.A., Manuel L.O. (2022). Schwinger boson theory of ordered magnets. Phys. Rev. B.

[66] Ghioldi E.A., Zhang S.S., Kamiya Y. (2022). Evidence of two-spinon bound states in the magnetic spectrum of Ba₃CoSb₂O₉. Phys. Rev. B.

[67] Zhu Z., White S.R. (2015). Spin liquid phase of the *S*=1/2 *J₁-J₂* Heisenberg model on the triangular lattice. Phys. Rev. B.

[68] Hu W.J., Gong S.S., Zhu W. (2015). Competing spin-liquid states in the spin-1/2 Heisenberg model on the triangular lattice. Phys. Rev. B.

[69] Ito S., Kurita N., Tanaka H. (2017). Structure of the magnetic excitations in the spin-1/2 triangular-lattice Heisenberg antiferromagnet Ba₃CoSb₂O₉. Nat. Commun..

[70] Jia H., Ma B., Wang Z.D. (2024). Quantum spin supersolid as a precursory Dirac spin liquid in a triangular lattice antiferromagnet. Phys. Rev. Res..

[71] Xie T., Eberharter A.A., Xing J. (2023). Complete field-induced spectral response of the spin-1/2 triangular-lattice antiferromagnet CsYbSe₂. npj Quantum Mater..

[72] Scheie A.O., Ghioldi E.A., Xing J. (2023). Proximate spin liquid and fractionalization in the triangular antiferromagnet KYbSe₂. Nat. Phys..

[73] Shu M., Dong W., Jiao J. (2023). Static and dynamical properties of the spin-5/2 nearly ideal triangular lattice antiferromagnet Ba₃MnSb₂O₉. Phys. Rev. B.

[74] Nakajima K., Ohira-Kawamura S., Kikuchi T. (2011). AMATERAS: A Cold-Neutron Disk Chopper Spectrometer. J. Phys. Soc. Jpn..

[75] Inamura Y., Nakatani T., Suzuki J. (2013). Development Status of Software “Utsusemi” for Chopper Spectrometers at MLF, J-PARC. J. Phys. Soc. Jpn..

[76] Azuah R.T., Kneller L.R., Qiu Y. (2009). Dave: A compressive software suite for the reduction, visualization, and analysis of low energy neutron spectroscopic data. J. Res. Natl. Inst. Stand. Technol..

