# Supplementary material for: Continuum of spin excitations in an ordered magnet
Source: Innovation (Camb). 2025 Jan 17;6(4):100769. doi: 10.1016/j.xinn.2024.100769 (PMC12131017; doi:10.1016/j.xinn.2024.100769)
Supplement: Document S1. Figures S1–S7 and supplemental materials and methods [file mmc1.pdf]

The Innovation, Volume *6*

Supplemental Information

Continuum of spin excitations in an ordered magnet

Jieming Sheng, Le Wang, Wenrui Jiang, Han Ge, Nan Zhao, Tiantian Li, Maiko Kofu, Dehong Yu, Wei Zhu, Jia-Wei Mei, Zhentao Wang, and Liusuo Wu

SUPPLEMENTAL INFORMATION

Linear spin wave

The semiclassical ground states of the TL XXZ model contain 3 sublattices in the magnetic unit cell for $B < B_s$, which we denote as $S\{\vec{s}_1, \vec{s}_2, \vec{s}_3\}$ where $|\vec{s}_i| = 1$ and $S \equiv 1/2$. Such $\sqrt{3}\times\sqrt{3}$ magnetic structures form a superlattice with a real-space basis $\boldsymbol{A}_1 = 2\boldsymbol{a}_1 + \boldsymbol{a}_2$ and $\boldsymbol{A}_2 = \boldsymbol{a}_1 + 2\boldsymbol{a}_2$, where $\{\boldsymbol{a}_1, \boldsymbol{a}_2\}$ is the basis of the original lattice.

Minimization of the classical energy at $T = 0$ with respect to $\{\vec{s}_1, \vec{s}_2, \vec{s}_3\}$ indeed reveals 3 phases below saturation. At intermediate field, we have $-\vec{s}_1 = \vec{s}_2 = \vec{s}_3 = (0,0,1)$, namely the UUD phase.

At low field, the optimal classical spin configuration is the "Y" phase:

$$\begin{aligned}\vec{s}_1 &= (0,0,-1),\\ \vec{s}_2 &= (\sin\theta, 0, \cos\theta),\\ \vec{s}_3 &= (-\sin\theta, 0, \cos\theta),\end{aligned} \tag{S1}$$

up to a global $U(1)$ rotation around the z-axis. The optimal value is

$$\cos\theta = \frac{\Delta + g_c\mu_B B/(3JS)}{\Delta+1}. \tag{S2}$$

The high-field "V" phase is:

$$\begin{aligned}\vec{s}_1 &= (-\sin\theta_1, 0, \cos\theta_1),\\ \vec{s}_2 = \vec{s}_3 &= (\sin\theta_2, 0, \cos\theta_2),\end{aligned} \tag{S3}$$

up to a global $U(1)$ rotation around the z-axis. The optimal values of $\{\theta_1\ \theta_2\}$ are obtained by numerically minimizing the classical energy in this report.

To perform the LSW, we first rotate to a local frame where the spins point to the $\hat{z} \equiv (0,0,1)$ direction:

$$\vec{s}_i = R_i\hat{z}, \tag{S4}$$

where R_i defines the SO(3) rotation matrix on each site. Accordingly, the spin operators transform as

$$\boldsymbol{S}_i = R_i\tilde{\boldsymbol{S}}_i. \tag{S5}$$

By representing the spin operators $\tilde{\boldsymbol{S}}_i$ with Holstein-Primakoff bosons:

$$\tilde{S}_i^+ = \sqrt{2S - b_i^\dagger b_i}\, b_i, \tag{S6a}$$

$$\tilde{S}_i^- = b_i^\dagger \sqrt{2S - b_i^\dagger b_i}, \tag{S6b}$$

$$\tilde{S}_i^z = S - b_i^\dagger b_i, \tag{S6c}$$

and keep up to quadratic order in the bosonic operators, we obtain the following Hamiltonian in Fourier space:

$$\mathcal{H} \approx \sum_{\tilde{\boldsymbol{k}}}{}' \Psi_{\tilde{\boldsymbol{k}}}^\dagger H_{\mathrm{LSW}}(\tilde{\boldsymbol{k}})\Psi_{\tilde{\boldsymbol{k}}} + C, \tag{S7}$$

where C is a constant,

$$\Psi_{\tilde{\boldsymbol{k}}} \equiv \left(b_{\tilde{\boldsymbol{k}},\boldsymbol{d}_1}, b_{\tilde{\boldsymbol{k}},\boldsymbol{d}_2}, b_{\tilde{\boldsymbol{k}},\boldsymbol{d}_3}, b^\dagger_{-\tilde{\boldsymbol{k}},\boldsymbol{d}_1}, b^\dagger_{-\tilde{\boldsymbol{k}},\boldsymbol{d}_2}, b^\dagger_{-\tilde{\boldsymbol{k}},\boldsymbol{d}_3}\right)^T, \tag{S8}$$

$H_{\rm LSW}(\tilde{\boldsymbol{k}})$ is a 6×6 Hermitian matrix, and the prime on the summation denotes that we are only summing over half of the folded Brillouin zone. Note that the presence of a superlattice requires a compatible Fourier transformation:

$$b_i \equiv b_{\tilde{\boldsymbol{r}}+\boldsymbol{d}} = \sqrt{\frac{3}{N}} \sum_{\tilde{\boldsymbol{k}}} e^{i\tilde{\boldsymbol{k}}\cdot\tilde{\boldsymbol{r}}} b_{\tilde{\boldsymbol{k}},\boldsymbol{d}}, \tag{S9}$$

where $\tilde{\boldsymbol{r}} = m\boldsymbol{A}_1 + n\boldsymbol{A}_2$ are positions of the superlattice, $\boldsymbol{d} = \{\boldsymbol{d}_1, \boldsymbol{d}_2, \boldsymbol{d}_3\}$ are positions of the 3 sublattices, and $N \rightarrow \infty$ is the total number of lattice sites. Note that $\tilde{\boldsymbol{k}}$ is related to the original momentum $\boldsymbol{k}$ by $\boldsymbol{k} = \tilde{\boldsymbol{k}} + \boldsymbol{K}$, where $\boldsymbol{K}$ denotes lattice points of the reciprocal superlattice.

The LSW Hamiltonian can be diagonalized by the Bogoliubov transformation: [1]

$$\Psi_{\tilde{\boldsymbol{k}}} = V_{\tilde{\boldsymbol{k}}} \tilde{\Psi}_{\tilde{\boldsymbol{k}}}, \tag{S10}$$

$$V^{\dagger}_{\tilde{\boldsymbol{k}}} H_{\rm LSW}(\tilde{\boldsymbol{k}}) V_{\tilde{\boldsymbol{k}}} = \mathrm{diag}\{\omega_{\tilde{\boldsymbol{k}},3}, \omega_{\tilde{\boldsymbol{k}},2}, \omega_{\tilde{\boldsymbol{k}},1}, \omega_{-\tilde{\boldsymbol{k}},1}, \omega_{-\tilde{\boldsymbol{k}},2}, \omega_{-\tilde{\boldsymbol{k}},3}\}. \tag{S11}$$

The dynamic spin structure factor including both the transverse (1-magnon) and longitudinal (2-magnon continuum) modes:

$$\begin{aligned} \mathcal{S}^{ab}(\boldsymbol{k},\omega) = {} & \pi S \sum_{j=1}^{3} A^a_j(\boldsymbol{k}) \left[A^b_j(\boldsymbol{k})\right]^* \delta\left(\omega - \omega_{\tilde{\boldsymbol{k}},j}\right) \\ & + 2\pi \int \frac{\mathrm{d}\tilde{\boldsymbol{q}}}{\mathcal{A}_{\rm BZ}} \sum_{j_1,j_2=1}^{3} A^a_{j_1 j_2}(\boldsymbol{k},\tilde{\boldsymbol{q}}) \left[A^b_{j_1 j_2}(\boldsymbol{k},\tilde{\boldsymbol{q}})\right]^* \\ & \cdot \delta(\omega - \omega_{-\tilde{\boldsymbol{q}},j_1} - \omega_{\widetilde{\boldsymbol{q}+\boldsymbol{k}},j_2}), \end{aligned} \tag{S12}$$

where $\mathcal{A}_{\rm BZ}$ is the area of the folded Brillouin zone, and

$$\vec{A}_j(\boldsymbol{k}) \equiv \frac{1}{\sqrt{3}} \sum_{l=1}^{3} e^{-i\boldsymbol{k}\cdot\boldsymbol{d}_l} R_l \begin{pmatrix} (V_{\tilde{\boldsymbol{k}}})_{l,4-j} + (V_{\tilde{\boldsymbol{k}}})_{l+3,4-j} \\ \left((V_{\tilde{\boldsymbol{k}}})_{l,4-j} - (V_{\tilde{\boldsymbol{k}}})_{l+3,4-j}\right)/i \\ 0 \end{pmatrix}, \tag{S13a}$$

$$\begin{aligned} \vec{A}_{j_1 j_2}(\boldsymbol{k},\tilde{\boldsymbol{q}}) \equiv {} & \frac{1}{\sqrt{3}} \sum_{l=1}^{3} e^{-i\boldsymbol{k}\cdot\boldsymbol{d}_l} \Big[(V_{-\tilde{\boldsymbol{q}}})_{l,4-j_1} \left(V_{\widetilde{\boldsymbol{q}+\boldsymbol{k}}}\right)_{l+3,4-j_2} \\ & + (V_{-\tilde{\boldsymbol{q}}})_{l+3,4-j_1} \left(V_{\widetilde{\boldsymbol{q}+\boldsymbol{k}}}\right)_{l,4-j_2} \Big] \boldsymbol{s}_l. \end{aligned} \tag{S13b}$$

The dynamic spin structure factor $\mathcal{S}(\boldsymbol{k},\omega)$ shown in this paper is defined as

$$\mathcal{S}(\boldsymbol{k},\omega) \equiv \sum_{a=x,y,z} \mathcal{S}^{aa}(\boldsymbol{k},\omega), \tag{S14}$$

and we have approximated the delta functions by Gaussian form with standard deviation $\sigma = 0.015\,\mathrm{meV}$.

Density matrix renormalization group

The dynamic spin structure factor is also calculated by the high-accuracy density matrix renormalization group (DMRG), which serves as an unbiased numerical solution to the current

problem. Cylindrical geometry is used in the DMRG calculation, with a periodic boundary in the y direction and an open boundary in the x direction. We denote it as $L_x \times L_y$ ($L_x \gg L_y$), where L_x and L_y are the number of unit cells in the x and y directions. We first obtain the ground state by optimizing the matrix product states on the whole cylinder and then target the dynamical properties (see below) by sweeping the middle $L_y \times L_y$ unit cells to avoid the boundary effect. This setup is equivalent to cutting the middle $L_y \times L_y$ unit cells and gluing them into a torus (with periodic boundary condition along both x- and y-directions), so that the momentum quantum number can be (approximately) defined along both x- and y-directions (within the $L_y \times L_y$ unit cells in the middle of the cylinder).

To calculate the dynamic spin structure factor, we need to target the following states together with the ground state $|0\rangle$ in the DMRG optimization process:

$$|S^\alpha(\boldsymbol{k})\rangle = S^\alpha(\boldsymbol{k})\,|0\rangle, \tag{S15a}$$

$$|x^\alpha(\omega + i\eta)\rangle = \frac{1}{\omega + i\eta - (H - E_0)}\,|S^\alpha(\boldsymbol{k})\rangle, \tag{S15b}$$

where $|x(\omega)\rangle$ is usually called the *correction vector* which can be calculated by the conjugate gradient method[2] or other algorithm.[3] Using the correction vector, the dynamic spin structure factor can be calculated through:

$$\mathcal{S}^{\alpha\beta}(\boldsymbol{k},\omega) = -\frac{1}{\pi}\mathrm{Im}\langle S^\alpha(\boldsymbol{k})|x^\beta(\omega + i\eta)\rangle, \tag{S16}$$

where the smearing energy is set to be $\eta = 0.2J$ in the calculation. In this work, the calculations were performed on $L_y = 6$ cylinders, and we ensure the truncation error is of the order or smaller than 10^{-5}, by keeping up to 2400 states in the DMRG process.

Additional spin excitation spectra

Figure S1 is the first measurement showing the spin excitation continuum of $Na_2BaCo(PO_4)_2$ along the high symmetry direction at $T = 50\,\mathrm{mK}$ and $B = 0\,\mathrm{T}$ using the time-of-flight cold neutron spectrometer, PELICAN, at the OPAL reactor, ANSTO, with $E_i = 3.7\,\mathrm{meV}$ and $0.13\,\mathrm{meV}$ energy resolution. To confirm the continuum nature, we performed further measurements with a better energy resolution on AMATERAS at J-PARC.

Figure S2 presents the inelastic neutron scattering (INS) spectra of $Na_2BaCo(PO_4)_2$ along the $[0,0,L]$ direction at high symmetry points (Γ, M, and K) measured at different magnetic fields and $T = 60\,\mathrm{mK}$ with field applied along the $\boldsymbol{c}$-axis. Below the saturation field B_s, the spin excitation spectra exhibit pronounced diffusion, particularly evident in the zero field where a broad continuum is observed. In contrast, for $B > B_\mathrm{s}$, the spectra manifest themselves as clean and sharply defined spin-wave excitations. Overall, the magnetic excitations along the $[0,0,L]$ direction remain nearly flat across all spectra, confirming the quasi-2D nature of the compound.

Figure S3 displays the spin excitation spectra of $Na_2BaCo(PO_4)_2$ along different high-symmetry momentum directions measured at $T = 450\,\mathrm{mK}$ (above T_N) and $B = 0\,\mathrm{T}$, $0.75\,\mathrm{T}$, respectively. The excitation spectra below and above T_N are similar at zero field, both manifesting broad continuum excitations, as illustrated in Fig. 4**A**-**B** and Fig. S3**A**-**B**. However, in the UUD phase ($B = 0.75\,\mathrm{T}$), there is a noticeable difference in the excitation spectra for temperatures below and above T_N. The initially sharp 1-magnon excitation in the UUD phase undergoes rapid diffusion as the temperature

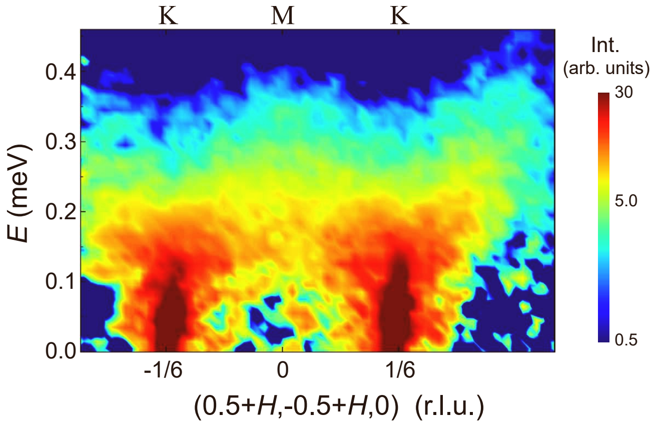

Figure S1: Spin excitation spectrum of $Na_2BaCo(PO_4)_2$ measured at $T = 50\,\text{mK}$ and $B = 0\,\text{T}$ along the high symmetry direction using the time-of-flight cold neutron spectrometer, PELICAN, at the OPAL reactor, ANSTO, with a fixed incident energy $E_\text{i} = 2.63\,\text{meV}$. The energy resolution is about $0.13\,\text{meV}$.

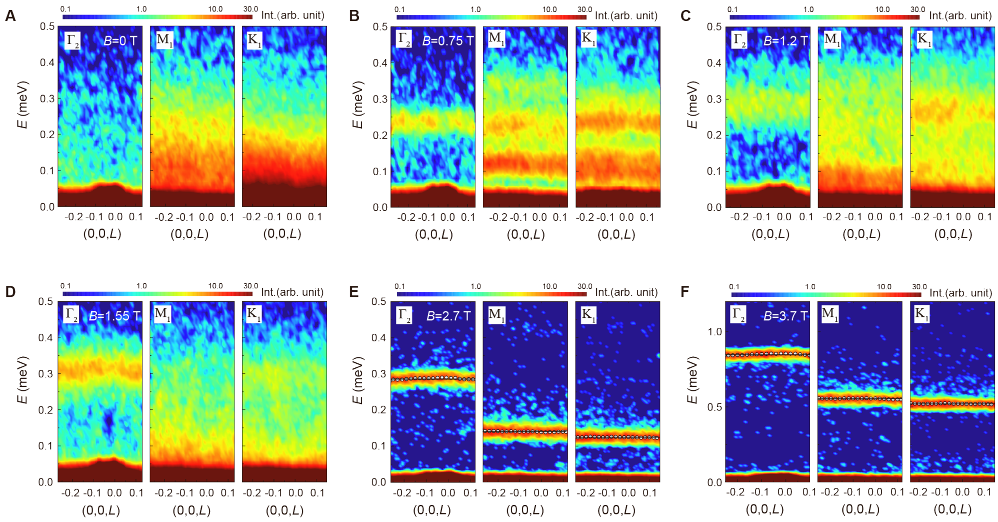

Figure S2: Inelastic neutron excitation spectra of $Na_2BaCo(PO_4)_2$ along the (0,0,L) direction at high symmetry points (Γ, M, and K) measured at $T = 60\,\text{mK}$ and **A** $B = 0\,\text{T}$, **B** $B = 0.75\,\text{T}$, **C** $B = 1.20\,\text{T}$, **D** $B = 1.55\,\text{T}$, **E** $B = 2.70\,\text{T}$, **F** $B = 3.70\,\text{T}$. Error bars denote standard deviations in **E** and **F**.

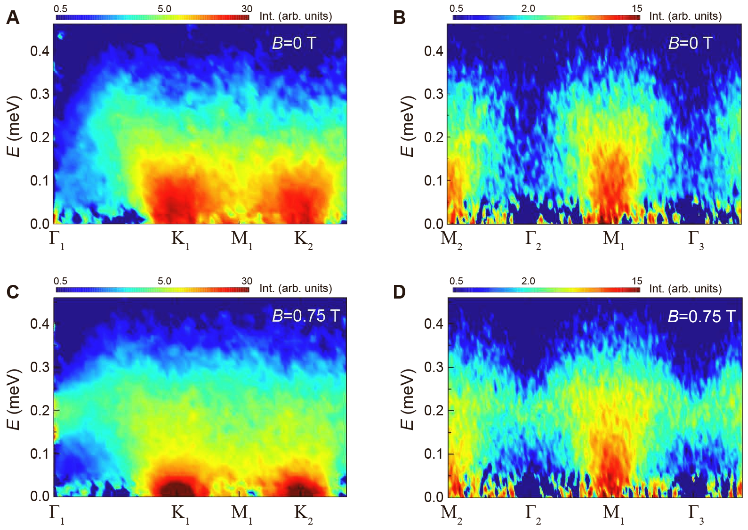

Figure S3: Spin excitation spectrum of $Na_2BaCo(PO_4)_2$ measured at $T = 450\,\text{mK}$ along the different high symmetry directions. **A** Γ_1-K_1-M_1-K_2 at $B = 0\,\text{T}$; **B** M_2-Γ_2-M_1-Γ_3 at $B = 0\,\text{T}$; **C** Γ_1-K_1-M_1-K_2 at $B = 0.75\,\text{T}$; **D** M_2-Γ_2-M_1-Γ_3 at $B = 0.75\,\text{T}$ using incident neutron energy $E_i = 2.63\,\text{meV}$. The intensity is integrated over the window of $L = [-0.2, 0.2]$. All the data was subtracted by a background of 60 mK-3.7 T data set.

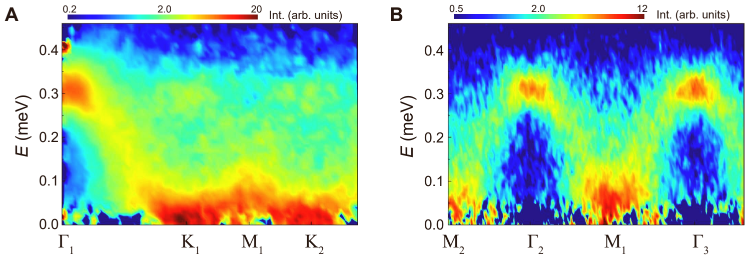

Figure S4: Spin excitation spectra of $Na_2BaCo(PO_4)_2$ measured at $T = 60\,\text{mK}$ and $B = 1.55\,\text{T}$ along the different high symmetry directions **A** Γ_1-K_1-M_1-K_2; **B** M_2-Γ_2-M_1-Γ_3 using incident neutron energy $E_i = 2.63\,\text{meV}$. The intensity is integrated over the window of $L = [-0.2, 0.2]$. The data was subtracted by a background of 60 mK-3.7 T data set.

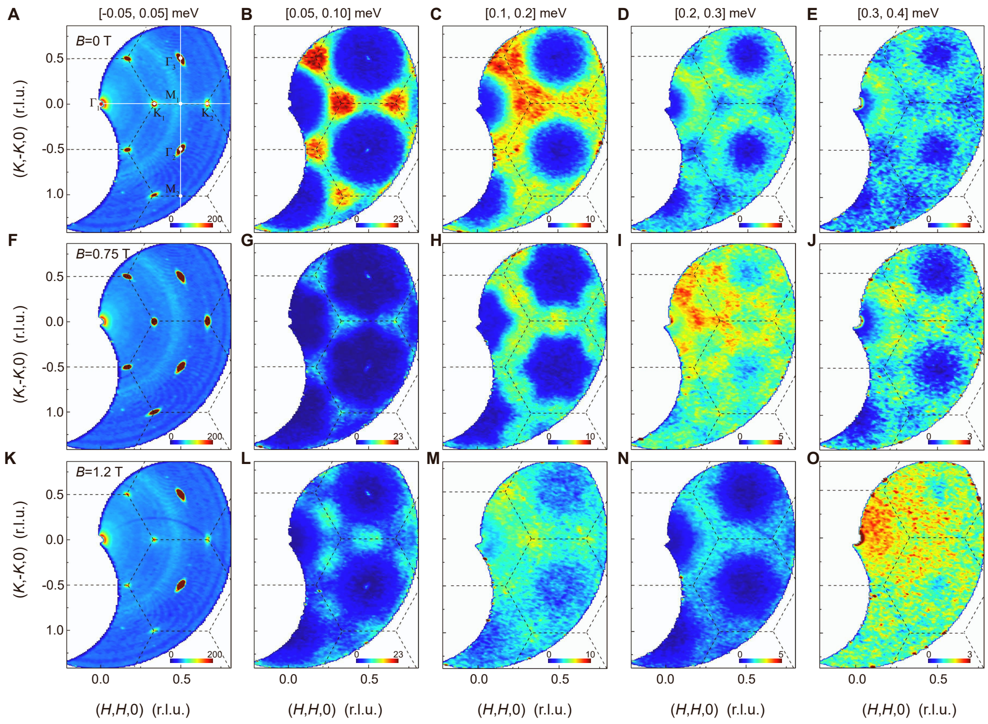

Figure S5: Measured momentum dependence of spin excitations in $Na_2BaCo(PO_4)_2$ at different constant energies $E = 0, 0.075, 0.15, 0.25, 0.35$ meV measured at $T = 60$ mK and **A**-**E** $B = 0$ T, **F**-**J** $B = 0.75$ T, **K**-**O** $B = 1.2$ T. The dashed lines indicate the zone boundaries.

crosses T_N, as depicted in Fig. 2**A**-**B** and Fig. S3**C**-**D**. This is a natural consequence due to breakdown of long-range magnetic order by thermal fluctuations.

Figure S4 shows the spin excitation spectra of $Na_2BaCo(PO_4)_2$ measured at $T = 60$ mK and $B = 1.55$ T along the different high symmetry directions. In addition to the strong 1-magnon spin wave, we observed weak scattering intensity that is broadly distributed across the energy-momentum plane.

Figure S5 are the measured momentum dependence of spin excitations of $Na_2BaCo(PO_4)_2$ at different constant energies with $T = 60$ mK and $B = 0$ T, 0.75 T, 1.2 T, respectively. The evolution of the scattering intensity with increasing energy is clearly observed in these figures. At zero energy, the magnetic Bragg peaks emerge at the K points for all the magnetic fields. At low energy ($E = 0.075$ meV) , strong scattering occurs around the K points for $B = 0$ T, while weak intensity is observed around the M points for $B = 1.2$ T and the K points for $B = 0.75$ T due to a gap opening at the K points around 0.05 meV, as illustrated in Fig. 2**A** in the manuscript. As energy increases, the position of strong scattering gradually evolves, and magnetic excitations become increasingly diffusive at high energy due to contribution from the two-magnon continuum.

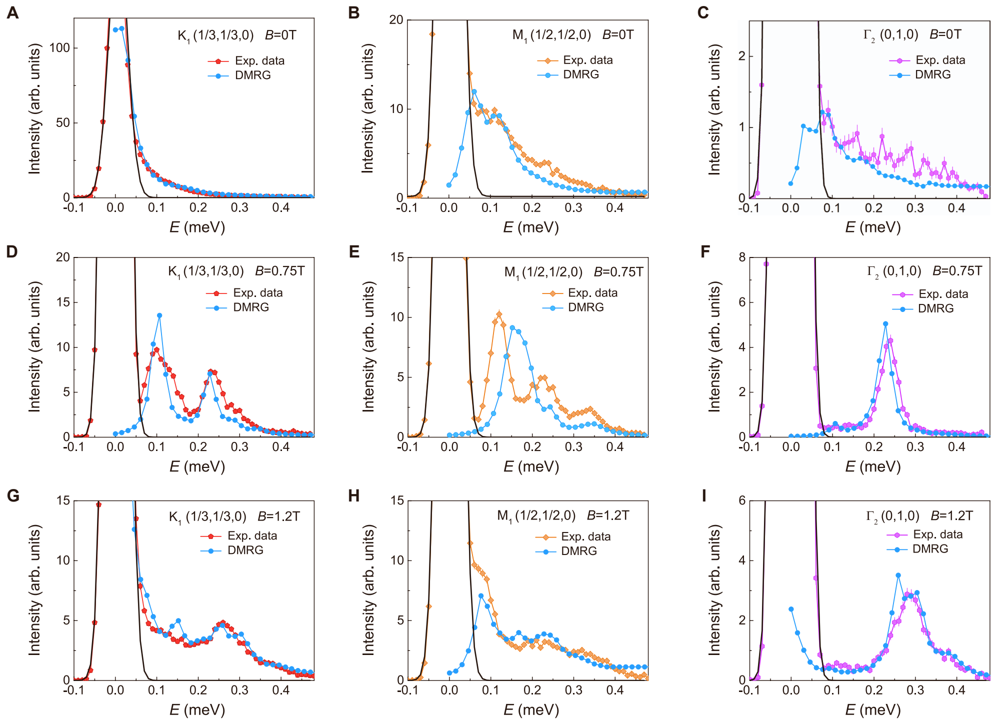

Figure S6: Comparison of the energy cuts between experiments and density matrix renormalization group (DMRG) calculations at high-symmetry points, K_1, M_1, Γ_2, with $T = 60\,\text{mK}$ and **A**-**C** $B = 0\,\text{T}$, **D**-**F** $B = 0.75\,\text{T}$, **G**-**I** $B = 1.2\,\text{T}$, respectively. The black solid lines represent Gaussian function fits to the elastic line. Error bars denote standard deviations.

Static spin structure factor calculated by DMRG

Figure S6 shows the energy dependence of spin excitations at high-symmetry points, K_1, M_1, Γ_2, under various magnetic fields $B = 0\,\text{T}$, $0.75\,\text{T}$, $1.2\,\text{T}$ at $T = 60\,\text{mK}$. According to the comparison of experimental data and DMRG calculations, it is clear that the easy-axis XXZ model captures the experimentally observed INS spectra almost quantitatively.

Figure S7 presents the $T = 0$ static spin structure factor of the 'Y', UUD and 'V' phases calculated by DMRG. In the 'Y' and 'V' phases, due to the presence of large in-plane spin components, the in-plane static spin structure factor is larger than the out-of-plane one. In contrast, in the UUD phase, since the spins are fully aligned along the c-axis, the in-plane static spin structure factor is suppressed. Additionally, as the magnetic field gradually increases, a portion of the spins become polarized, which results in the magnetic Bragg peaks emerging at the Γ points in the UUD and 'V' phases. When the spins are fully polarized, the static spin structure factor at the K points is completely suppressed, with only the magnetic Bragg peaks appearing at the Γ points.

References

[1] J. H. P. Colpa, Diagonalization of the quadratic boson hamiltonian, Physica A 93 (1978) 327–353. doi:10.1016/0378-4371(78)90160-7.

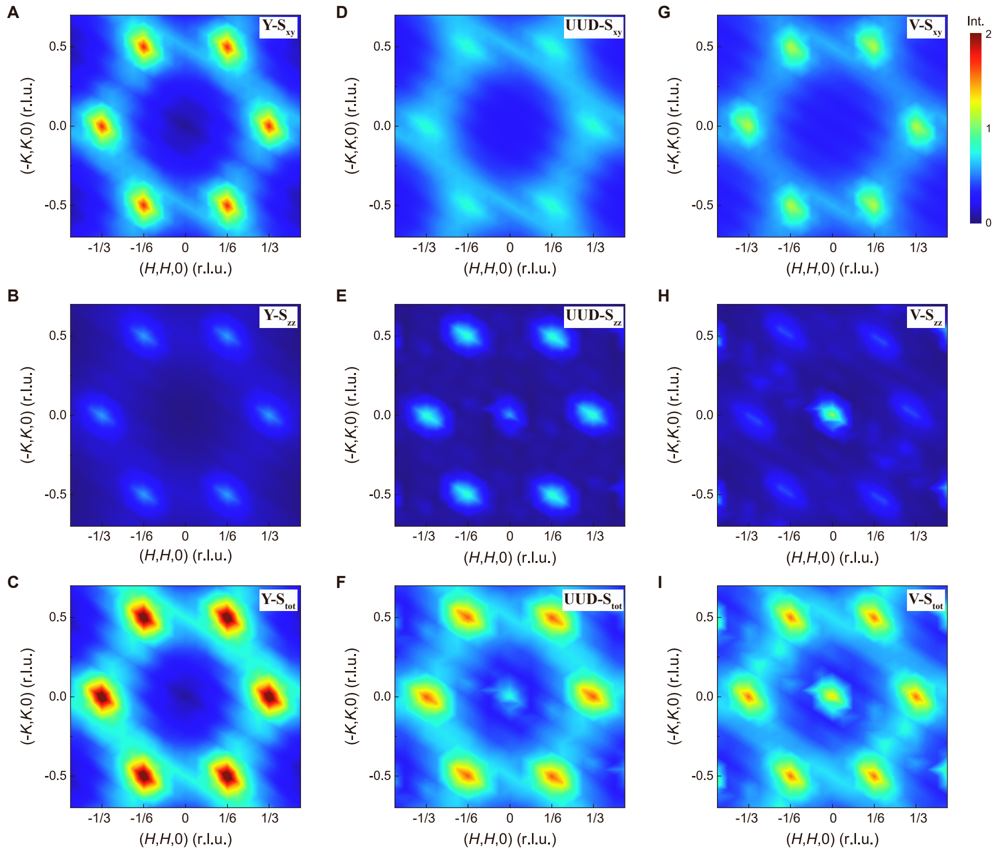

Figure S7: $T = 0$ static spin structure factor of the 'Y' ($B = 0\,\mathrm{T}$), UUD ($B = 0.75\,\mathrm{T}$) and 'V' ($B = 1.2\,\mathrm{T}$) phases calculated by DMRG on 6-leg cylinder. **A**-**C** are the in-plane, out-of-plane and total static spin structure factors of the 'Y' phase, respectively. **D**-**F** are the in-plane, out-of-plane and total static spin structure factors of the UUD phase, respectively. **G**-**I** are the in-plane, out-of-plane and total static spin structure factors of the 'V' phase, respectively.

[2] T. D. Kühner, S. R. White, Dynamical correlation functions using the density matrix renormalization group, Phys. Rev. B 60 (1999) 335–343. doi:10.1103/PhysRevB.60.335.
[3] E. Jeckelmann, Dynamical density-matrix renormalization-group method, Phys. Rev. B 66 (2002) 045114. doi:10.1103/PhysRevB.66.045114.
